# Pleural mesothelioma and venous thrombosis: the eosinophilia link

**DOI:** 10.1186/1477-9560-6-3

**Published:** 2008-04-28

**Authors:** Paul Richard Julian Ames, Win Win Aye

**Affiliations:** 1 Department of Haematology, West Suffolk Hospital, Hardwick Lane, Bury St Edmunds, IP33 2QZ, UK

## Abstract

Peripheral blood eosinophilia and vascular occlusions are rare occurrences in patients with pleural mesothelioma whereas eosinophilia may associate with thrombosis. We describe a patient with mesothelioma who developed peripheral blood eosinophilia followed by deep vein thrombosis despite being on low molecular weight heparin prophylaxis. We discuss the genesis of peripheral blood eosinophilia and thrombosis in pleural mesothelioma.

## Introduction

The latest nationwide census from the occupational United Kingdom physicians identified pleural mesothelioma (PM) in 92% of lung cancers patients [1] whereas PM represented only 8% of histology proven lung cancers accrued from an area without heavy industrial exposure to asbestos [2]. Peripheral blood eosinophilia (PBE) has been detected in 0.5% to 1.7% of patients with malignancy [3]: it may occur in cancer of the lung, pancreas, uterus, liver, breast, kidney and thyroid [3,4], in liposarcomas [5] in lymphomas and T-cell-leukemias [6,7] but it is quite rare in PM [8-11]. PBE is associated with metastatic spread, predicts a poor prognosis [12,13], may disappear with treatment of the malignancy and reappear with tumour relapse [6,14]. We describe a patient who developed PBE almost coincidentally with PM followed by deep vein thrombosis despite low molecular weight heparin prophylaxis and in the absence of inherited thrombophilia.

## Case report

A 75-year old gentleman was admitted to the Oncology/Haematology Ward in early September 2007 for severe shortness of breath. He had been fully investigated for a pleural effusion one month earlier in a neighbouring hospital. At that time drained pleural fluid revealed reactive lymphocytes with neither neutrophils nor eosinophils. However peripheral blood esosinophils were raised at 1.6 × 10^9^/L, C-reactive protein (CRP) at 33 mg/dl and fibrinogen at 568 mg/dl. Cultures for bacteria and stains for acid-fast bacilli were negative. Several stool examinations and serologic tests were negative for parasites as well as an autoimmune screen (antinuclear antibodies, antineutrophil-cytoplasmic antibodies, anti myeloperoxidase antibodies). A right pleural biopsy done in mid August 2007 revealed sheets of pleura infiltrated by malignant epithelioid cells that on immune staining expressed CK7 (cytokeratin) with focal expression of CK5/6 whereas staining for carcino-embriogenic antigen, BerEP4, thyroid transcription factor-1 mesothelin, Wilms' tumour -1, CK20 or prostate specific antigen was negative, confirming a diagnosis of epithelioid mesothelioma. A drain was inserted through the right chest wall and opiates started (morphine sulphate 30 modified release bd, oxycodone). During the current admission the drain was re-sited and fluid examination revealed only reactive lymphocytes and few neutrophils. His peripheral blood eosinophils had risen to 21 × 10^9^/L, CRP was up at 88 mg/dl and fibrinogen at 880 mg/dl. The patient was started on a prophylactic dose of tinzaparin (3500 IU/day). On the fourth day after admission the patient developed a swollen calf, his D-dimer was greater than 1000 mg/dl (cut-off normal >280 mg/dl) and an ultrasound scan confirmed a fresh popliteal vein thrombosis. Tinzaparin was increased to therapeutic levels for five days until warfarin brought the international normalised ratio within therapeutic range. A thrombophilia screen (including factor V Leiden, prothrombin 20210, protein C, protein S, antithrombin, lupus anticoagulant and anticardiolipin antibodies) arranged before commencing warfarin came back normal. Neither the FIP1L1-PDGFRA fusion transcript (deletion 4q12) nor other cytogenetic abnormalities were found on a cellular sample of peripheral blood. After 5 days later his general conditions deteriorated, he became progressively confused with increasing hypoxia and passed away for respiratory failure. Post mortem revealed a mesothelioma that had diffusely surrounded and invaded the left lung and pericardium without directly affecting vascular structures. No asbestos bodies were found and no pulmonary embolism was detected.

## Discussion

PBE is rare finding in mesothelioma: a large survey found one case out of 4710 patients with pleural involvement [8] whereas two more patients with pleural involvement [9,10] and one with peritoneal involvement [11] have been described as case reports. In this setting PBE should be considered reactive as the tumour itself produces factors that promote eosinophil generation such as interleukin 3, interleukin 5 and granulocyte-monocyte colony stimulating factor [15]. The negative cytogenetic testing favours a reactive PBE in our patient.

Thrombosis development in PM is also rare: according to one series venous occlusions occurred in 6.5% (1/15) of cases [16], whereas an autopsy study on 111 patients with lung cancer identified 14 cases of PM of which 14% had evidence of clots in the venous circulation [17]; isolated reports documented occlusions of the superior vena cava [18,19], of the left jugular and subclavian veins [20] and of the portal vein [21]. Arterial occlusions affected cerebral circulation in two cases [22,23] and mesenteric vessels in one case (associated with nephrotic syndrome)[24].

The pathogenesis of thrombosis in PM is partly due to tumour tissue compressing veins [18,20] that at times may progress to direct vessel infiltration [19] and partly due to a hypercoagulable state characterised by elevated levels of plasma fibrinogen [25] and increased fibrin turnover leading to disseminated intravascular coagulation [26].

Primary eosinophilic syndrome is strongly associated with thrombosis that may be recurrent despite adequate heparin and warfarin anticoagulation [27]. Eosinophils store and express tissue factor and once activated may initiate coagulation [28]. In addition eosinophils interfere with the activity of natural anticoagulants: eosinophilic cationic protein (ECP) binding to thrombomodulin prevents thrombin dependent activation of anticoagulant protein C [29] and binding to heparan sulphate (or heparin) prevents the conformational change required for the anti-thrombin neutralisation of thrombin [30]. Indeed an excess of ECP was able to override heparin binding to antithrombin [31] and may well explain the few instances where apparently therapeutic or prophylactic doses of heparin failed to prevent thrombosis [27,32]. This was the situation of our patient who developed deep vein thrombosis being already on prophylactic low molecular weight heparin.

In conclusion we have reported an unprecedented patient with pleural mesothelioma in whom reactive PBE contributed to the development of deep vein thrombosis more than the tumour itself on the background of an inflammatory and eventually hypercoagulable state. Though difficult for mesothelioma, tumour treatment may relieve the reactive PBE [6,14]. Clinicians should be aware of the added thrombotic risk of hypereosinophilia in cancer patients [33] and consider prophylactic or therapeutic anticoagulation according to clinical judgement and laboratory guidance, possibly with anti factor Xa monitoring in the case of low molecular weight heparin.
